# Enhanced inhibition of Avian leukosis virus subgroup J replication by multi-target miRNAs

**DOI:** 10.1186/1743-422X-8-556

**Published:** 2011-12-22

**Authors:** Qing-Wen Meng, Zai-Ping Zhang, Wei Wang, Jin Tian, Zhi-Guang Xiao

**Affiliations:** 1 State Key Laboratory of Veterinary Biotechnology,Harbin Veterinary Research Institute, Chinese Academy of Agricultural Sciences, No.427 Maduan Street, Nangang District, Harbin 150001, People's Republic of China

**Keywords:** ALV, miRNA, Inhibition, Gag, Multi-target series

## Abstract

**Background:**

Avian leukosis virus (ALV) is a major infectious disease that impacts the poultry industry worldwide. Despite intensive efforts, no effective vaccine has been developed against ALV because of mutations that lead to resistant forms. Therefore, there is a dire need to develop antiviral agents for the treatment of ALV infections and RNA interference (RNAi) is considered an effective antiviral strategy.

**Results:**

In this study, the avian leukosis virus subgroup J (ALV-J) proviral genome, including the *gag *genes, were treated as targets for RNAi. Four pairs of miRNA sequences were designed and synthesized that targeted different regions of the *gag *gene. The screened target (i.e., the *gag *genes) was shown to effectively suppress the replication of ALV-J by 19.0-77.3%. To avoid the generation of escape variants during virus infection, expression vectors of multi-target miRNAs were constructed using the multi-target serial strategy (against different regions of the *gag*, *pol*, and *env *genes). Multi-target miRNAs were shown to play a synergistic role in the inhibition of ALV-J replication, with an inhibition efficiency of viral replication ranging from 85.0-91.2%.

**Conclusion:**

The strategy of multi-target miRNAs might be an effective method for inhibiting ALV replication and the acquisition of resistant mutations.

## Background

Avian leukosis (AL) is the general term for a variety of neoplastic diseases of poultry caused by the *Alpharetrovirus*, Avien leukosis virus (ALV). ALV has been classified into 10 subgroups, designated A-J. The subgroup J virus (ALV-J) is a relatively new strain of ALV that was isolated from Dorking fowl in the early 1990s [1]. ALV is an RNA virus with a genome of approximately 7.6 kb. The proviral genome of ALV-J contains three major genes, *gag*, *pol*, and *env*, which encode the viral structural proteins, RNA-dependent DNA polymerase, and the envelope glycoprotein, respectively.

RNA interference (RNAi) is a simple and effective tool for silencing target genes that involves endogenous or exogenous double-stranded RNA (dsRNA)-mediated degradation of the specific mRNA sequences. The main nucleic acid molecules that induce gene silencing are small interfering RNA (siRNA) and microRNA (miRNA), where the siRNAs mediate specific mRNA degradation, whereas miRNA inhibits specific mRNA at the translational level. Both of these biological processes are considered key methods of modulating host gene expression, and these two molecules are also involved in antiviral and transposon silencing pathways.

The RNAi strategy has been successfully applied to the inhibition of viral replication. It has been demonstrated that some genes inhibited by siRNAs, such as *p24*, *vif*, *nef*, *tat*, and *rev*, can block Human immunodeficiency virus (HIV) replication in cells [2]. The infection of cells by HIV may be hindered by inhibiting the expression of the HIV receptors CD4 and CD8a, their coreceptors CXCR4 or CCR5, or the virus Gag structural protein [3]. In some studies, transfection of siRNA designed to target C virus (HCV) remarkably inhibited the expression of virus-specific proteins and protected cells against HCV RNA, *in vitro *[4,5]. In another study, Hepatitis B virus (HBV) replication was successfully inhibited after plasmid expression of HBV siRNA transfected into mouse liver [6]. Hu *et al. *[7] adopted siRNA designed against the ALV *gag *gene and demonstrated significantly reduced virus replication. Chen *et al. *[8] indicated that ALV-B replication was significantly inhibited after knockdown of the ALV-B *tvb *and *env *genes.

Although siRNAs have been widely used as gene-silencing molecules, the intrinsic drawbacks of siRNA methodology have been revealed. Off-target effects may be produced where siRNA function must be fully and completely complementary to the target sequence; if the virus mutates, siRNA will produce off-target effects. Other drawbacks include the elicitation of the interferon response and interference with endogenous miRNA biogenesis. The unique biogenesis and mechanism of action of miRNA do reduce the likelihood of these problems.

Despite these problems, the RNAi strategy remains an attractive option for antiviral therapy and for the functional analysis of genes for several reasons. First, RNAi has sequence specificity. Second, the application of multi-series RNAi can target different genes or sequences simultaneously and hence can minimize the possibility of the virus acquiring mutations that confer resistance. Third, RNAi can be transmitted from non-pathogenic viruses to pathogenic viruses. Finally, due to its sequence specificity, siRNA designed against a virus can only inhibit that virus, leaving vaccine strains unaffected. To ensure a high level of RNAi gene silencing, multiple miRNAs can be designed. These can be transfected into cells using the same transfer medium, and by targeting different sequences, mutation of the target virus, and therefore the probability of evasion from the silencing effect of the miRNA is minimized.

ALV, especially ALV-J, brings about enormous economic losses in the poultry industry. The virus has rapidly spread worldwide and transmits both vertically and horizontally. However, chicks are immunologically tolerant to ALV infection. Unfortunately, to date, no effective vaccine has been developed against ALV. In this study, we used RNAi technology to inhibit ALV replication and screened the effective target sites for their ability to inhibit ALV replication at a cellular level. Our findings could pave the way for anti-ALV gene screening and the development of disease resistance.

## Materials and methods

### Viruses and cells

The SD strains of ALV-J were isolated from poultry in Shandong, China, and stored at the Harbin Veterinary Research Institute (Harbin, China), a Key State Laboratory. The DF-1 cell lines [9] were provided by Zhigao Bu at the Harbin Veterinary Research Institute (Harbin, China).

### Reagents

The linearized the pcDNA6.2-GW/EmGFP-miR eukaryotic expression vector, *Escherichia coli *TOP10 cells and the Lipofectamine 2000 transfection reagent were purchased from Invitrogen (Carlsbad, CA, USA). Ligase and reverse transcriptase were purchased from TaKaRa (Dalian, China). The fluorescence quantitative PCR kit was purchased from BIOER Technology (Hangzhou, China). The plasmid extraction kit and the viral RNA extraction kit were purchased from Shanghai Watson Biotech (Shanghai, China). The goat anti-mouse IgG/fluorescein isothiocyanate (FITC) antibody and the horseradish peroxidase-labeled goat anti-mouse IgG were purchased from Zhongshan Goldbridge Biotechnology (Beijing, China). The anti-ALV-J monoclonal antibody JE-9 was kindly provided by Professor Qin Aijian (Yangzhou University, China). All other chemicals were of analytical reagent grade.

### Design and construction of miRNA expression vector

#### Design of miRNA

Four pairs of miRNAs sequences were designed against the conserved regions of the *gag *gene (NC-015116 gag) using online software http://rnaidesigner.invitrogen.com/rnaiexpress/ (Table 1). Designed miRNA sequences were synthesized by Shanghai Health Bioengineering (China). Double-stranded oligonucleotide encoding pre-miRNA sequence were annealed and inserted into the linearized expression vector, pcDNA6.2-GW/EmGFP-miR (5 ng/μL), to construct recombinant plasmids containing the target miRNAs, designated mi-*gag*1318, mi-*gag*1365, mi-*gag*1623, mi-*gag*1971. All recombinant plasmids have been sequenced to confirm the sequences inserted.

**Table 1:** Oligonucleotide sequences of pre-miRNAs

Sequence (5' to 3')	
mi-*gag*1318	TGCTGTTGATCACAAGACTGGCTGATGTTTTGGCCACTGACTGACATCAGCCACTTGTGATCAA
	
	CCTGTTGATCACAAGTGGCTGATGTCAGTCAGTGGCCAAAACATCAGCCAGTCTTGTGATCAAC

mi-*gag*1365	TGCTGTAGTGATTAAGACAGAGGGACGTTTTGGCCACTGACTGACGTCCCTCTCTTAATCACTA
	
	CCTGTAGTGATTAAGAGAGGGACGTCAGTCAGTGGCCAAAACGTCCCTCTGTCTTAATCACTAC

mi-*gag*1623	TGCTGATCATTGCGGAACAGCTATTGGTTTTGGCCACTGACTGACCAATAGCTTCCGCAATGAT
	
	CCTGATCATTGCGGAAGCTATTGGTCAGTCAGTGGCCAAAACCAATAGCTGTTCCGCAATGATC

mi-*gag*1971	TGCTGTTATGTCTCCCTCAGACTTATGTTTTGGCCACTGACTGACATAAGTCTGGGAGACATAA
	
	CCTGTTATGTCTCCCAGACTTATGCAGTCAGTGGCCAAAACATAAGTCTGAGGGAGACATAAC

**Table 2:** Primers used to amplify target genes

Target gene (Accession number)	Primer sequence	Product size (bp)	Annealing Temperature (°e)
*Gag*	Forward TCAGGACCAAGGGCTTAC	174	55.2
			
	Reverse CTGCCGCTATAACCGTCTG		

*ALV specific primer*	Forward TCAGGACCAAGGGCTTAC	545	55.0
			
	Reverse CTGCCGCTATAACCGTCTG		

β-*actin*	Forward TCCCTGTATGCCTCTGGTC	250	55.0
			
	Reverse TCTCTCTCGGCTGTGGTGG		

#### Construction of miRNA expression vectors in series

Two tandem plasmids were constructed based on the mi-*gag*1318 plasmid backbone. After *Bgl*I and *Xho*I digestion of mi-*gag*1318, the miRNA sequence of the *pol *gene (NC-015116 *pol*), mi-*pol*2516 (constructed previously, manuscript submitted), was digested with *Sal*I and *Bgl*I, and ligated to mi-*gag*1318. The tandem plasmid consisting of mi-*gag*1318 and mi-*pol*2516 was designated mi-g1318-p2516. The mi-g1318-e1384 (*env *gene: NC-015116 *env*) and mi-p2516-e1384 plasmids were constructed in the same way. Plasmids were linearized and used to transform *E. coli *TOP10. Positive clones were selected and verified by digestion with *Xho*I and *Sal*I and sequencing. Similarly, based on the recombinant plasmid mi-g1318-p2516 backbone, a tandem plasmid consisting of mi-*gag*1318, mi-*pol*2516 and mi-*env*1384 was constructed in the same way and designated mi-g1318-p2516-e1384. Positive clones were verified by double digestion with *Xho*I and *Sal*I and sequencing.

### Transfection of recombinant plasmid and preparation of ALV

DF-1 cells were cultured in Dulbecco's Modified Eagle's Medium (DMEM) containing 100 U/mL of penicillin and streptomycin, and containing 5% (v/v) fetal calf serum (PAA GOLD, Austria). Twenty-four hours before transfection, cells that had grown to 70% confluence were used to inoculate cell culture plates at a density of 3-5 × 10^5^. When the cells had grown to 80% confluence, they were transfected with the RNAi expression plasmid using Lipofectamine 2000. Six hours later, the culture fluid was changed DMEM and serum containing 5% fetal calf serum, without antibiotics. After incubation at 37°C and 5% CO_2 _for 12 h, the transfection efficiency was determined by the expression of green fluorescent protein observed using fluorescence microscopy. Transfected cells were inoculated with 100 50% tissue culture infectious doses (TCID_50_) of ALV-J. The culture medium was obtained 72 h after viral infection, and the inhibitory effect was detected by an indirect immunofluorescence assay (IFA), western blotting, and real-time PCR. In this study, the negative control group that was not administered plasmid, the null vector control group and the test groups were randomized for analysis.

### Determination of ALV-J titer

Strain SD was inoculated into DF-1 cell culture bottles, freeze-thawed three times and then centrifuged at 7000 × *g *and 4°C for 5 min. The supernatant was collected and 10-fold serially diluted to 10^-8 ^and then inoculated into 96-well plates along with DF-1 cells. Four parallel well were created in each gradient. After incubation for 5 days, cells were fixed by cooling in methanol at -20°C for 30 min, and then washed three times with phosphate buffered saline containing 0.05% Tween 20 (PBST) for 10 min each. ALV-J specific monoclonal antibody JE-9 was diluted 1:200 and 100 μL was added to each well. The cells were incubated at 37°C for 1 h and washed with PBST three times, for 10 min each. The secondary antibody, goat anti-mouse IgG/FITC, was added at a dilution of 1:200, and the cells were incubated at 37°C for 45 min away from the light. The cells were then washed with PBST five times, for 10 min each. The number of fluorescent cells per well were counted using a fluorescence microscope, and the TCID_50 _was calculated using the Reed-Muench method [10]. Therefore, the toxic potency of the virus was determined to be 10^5.2 ^TCID_50_/mL.

### Expression of the envelope glycoprotein by IFA

Virus strain SD was used to inoculate DF-1 cells transfected with recombinant plasmid using the method described above. After 5 days incubation, cells were treated with the fluorescent secondary antibody, rhodamine-labeled goat anti-mouse IgG/TRITC, as described above. The results were photographed using a fluorescence microscopy.

### Western blot

At about 72 h after transfection with the miRNA recombinant plasmid, DF-1 cells were collected and the residual supernatant fluid was washed with PBS. Total protein was extracted using the total protein extraction kit (BestBio, China) and quantitated using a spectrophotometer. Sodium dodecyl sulfate-polyacrylamide gel electrophoresis (SDS-PAGE; 10%) was performed using a sample volume of 300 μg per well. Samples were transferred to nitrocellulose membrane after separation, blocked at 4°C overnight and then washed with PBST three times, for 10 min each. ALV-J-specific monoclonal antibody JE-9 was added and the cells were incubated at 37°C for 1 h and mouse anti-chicken glyceraldehyde-3-phosphate dehydrogenase (GAPDH) monoclonal antibodies were added and incubated at room temperature for 1 h, followed by three washes with PBST for 10 min each. Horseradish peroxidase-labeled anti-mouse secondary antibody was added and cells were incubated at 37°C for 45 min. After washing with PBST five times, 10 min each wash, the cells were stained with 3,3' diaminobenzidine (DAB, 6 mg, 10 mL TBS, 0.1 mL 3% H_2_O_2_) and then scanned using the gel imaging system AlphaImager HP.

### Quantitative PCR

Using sequences of the ALV *gag *genes published in the GenBank database (NC-015116), pairs of primers were designed using the Oligo 6 primer design software to amplify the conserved regions of the gene. Additionally, a pair of ALV-J specific primers was also synthesized. Two pairs of target genes and the *β-actin *gene were amplified by PCR using the SD cDNA as a template (Table 2).

The cDNAs amplified using the *gag*, ALV-specific, and *β-actin *primers were cloned into vector pMD-18-T and used to transform competent *E. coli *DH5α cells. Positive clones were isolated and verified by colony PCR and sequencing, and designated pMT-G and pMT-ALV, respectively. The concentration and purity of verified plasmids were determined according to the formula: copy number = (mass/molecular weight) ×6.0 × 10^23^. Extracted plasmids were then ten-fold serially diluted and used as temples for quantitative PCR amplification and delineation of quantitative PCR standard curves.

Total RNA was extracted from viruses and cells 72 h after the infection of DF-1 cells with ALV-J using Trizol, according to the manufacturer's instructions. RNA concentration and purity was determined by measuring optical density (OD) at wavelengths of 260 and 280 nm using a standard spectrophotometer. The OD_260_/OD_280 _ratios were more than 1.8 for all samples. Quantitative PCR was performed in a LightCycler 480 Real-Time PCR System, for the *gag *and ALV-specific genes using the SYBR GREEN kit (SYBR^® ^Premix Ex Taq™, TaKaRa, Dalian, China) and the primers are listed in Table 1. Amplification was carried out in a 20 μl reaction mixture containing 10 μl SYBR^® ^Premix Ex Taq™2×, 0.2 μM concentration of each primer, 1 μl cDNA. The reaction procedure was 95°C 10 s, followed by 40 cycles at 95°Cfor 5 s and 60°C for 40 s. The number of target genes in different samples was determined according to the standard curve after the reaction. Meanwhile, the copy number of the *β-actin *gene was also determined by quantitative PCR. To confirm specific amplification, melting curve analysis was performed.

### Data analysis

Experiments were repeated three times and values were expressed as means ± standard deviation (SD). The *t*-test was performed using the SPSS 13.0 statistical software (version 13.0; SPSS, USA). Differences were considered statistically significant when *p *< 0.05.

## Results

### Envelope protein expression

**Figure 1:**
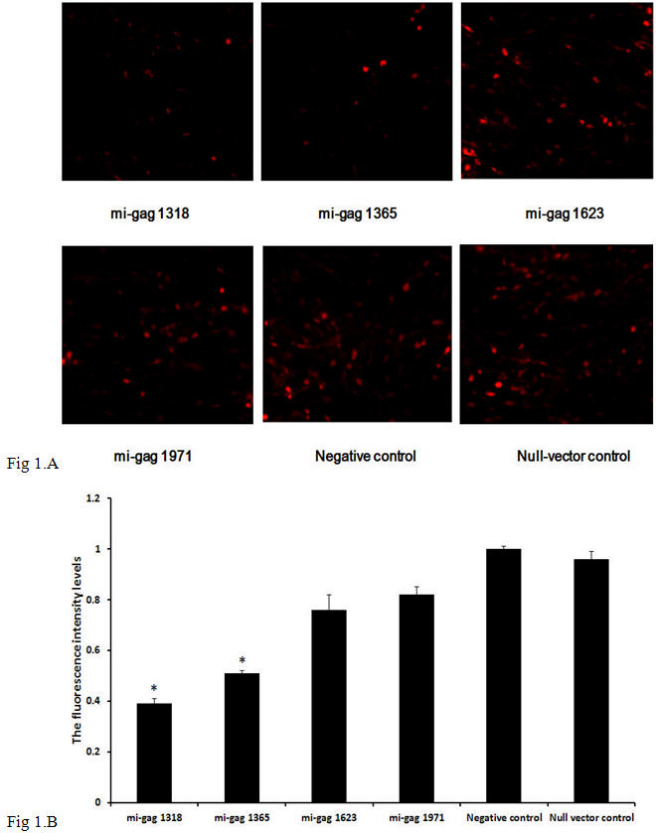
**ALV-J replication**. (A) Cells transfected with the recombinant plasmid pMD-G and their inhibitory effects against ALV-J, as determined by the IFA. (B) The fluorescence intensity of cells transfected with the recombinant plasmid pMD-G and their inhibitory effects against ALV-J, as determined by the IFA. Data are presented as means±S.E.M. of three independent experiments, each performed in triplicate. *Statistically significant differences compared with negative controls (P<0.05).

**Figure 2:**
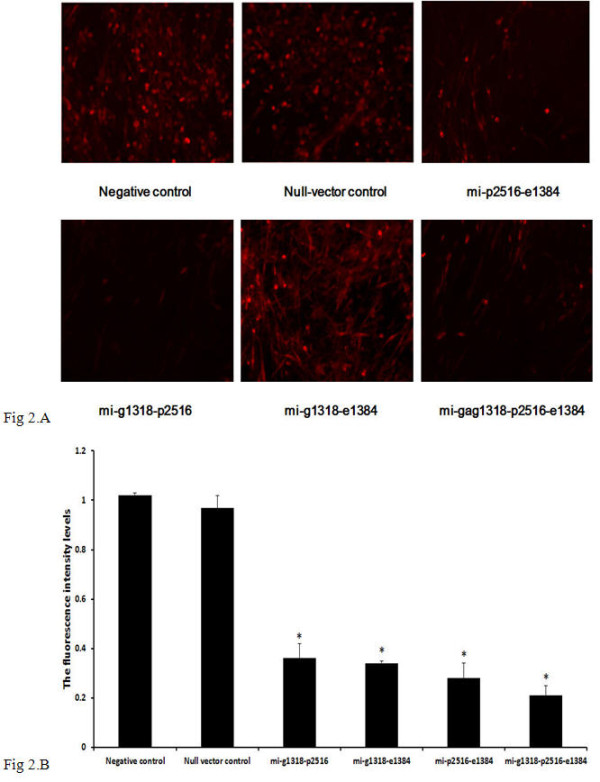
**Inhibition of ALV-J replication by miRNA**. (A) Cells transfected with the recombinant serial miRNA plasmids and their inhibitory effects against ALV-J, as determined by the IFA. (B) The fluorescence intensity of cells transfected with the recombinant serial miRNA plasmids and their inhibitory effects against ALV-J, as determined by the IFA. Data are presented as means±S.E.M. of three independent experiments, each performed with triplicate samples. *Statistically significant differences compared with negative controls (P<0.05).

As shown in Figure 1a and 1b, IFA revealed that the recombinant plasmids, mi-*gag*1318 and mi-*gag*1365, significantly reduced the fluorescence intensity. The tandem plasmids, mi-g1318-p2516, mi-g1318-e1384, mi-p2516-e1384 and mi-g1318-p2516-e1384, also significantly inhibited the fluorescence intensity (Figure 2a and 2b) compared with the negative control group. However, no significant differences were detected among other groups compared with the negative control group and the null vector group.

### Western blot analysis of the ALV-J envelope glycoprotein

**Figure 3:**
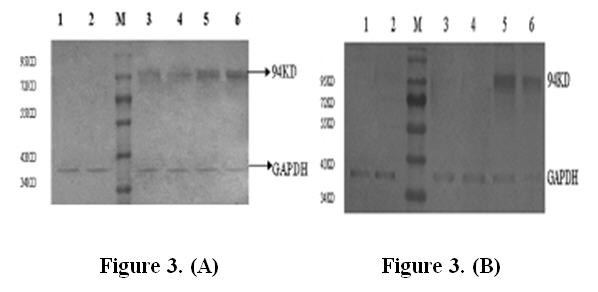
**ALV-J envelope glycoprotein expression of DF-1 cells in each in of the groups**. (A) Western immunoblot of infected culture treated with miRNA. Lane M: protein ladder; lane 1: mi-*gag*1318; lane 2: mi-*gag*1356; lane 3: mi-*gag*1623; lane 4: mi-*gag*1971; lane 5: negative control; lane 6: null vector control. (B) Western immunoblot of infected cultures treated with miRNA. Lane M: protein ladder; lane 1: mi-g1318-e1384; lane 2: mi-p2516-e1384; lane 3: mi-g1318-p2516; lane 4: mi-g1318-p2516-e1384; lane 5: negative control; lane 6: null vector control.

JE-9 is an anti-ALV-J envelope glycoprotein monoclonal antibody that specifically identifies a protein with a molecular weight of 90-94 kDa. As detected by western blot analysis (Figure 3a, b), the recombinant mi-*gag*1318 and mi-*gag*1365 plasmids decreased ALV-J envelope glycoprotein expression significantly, while there were no significant differences determined between the remaining groups and the negative control or null vector groups. Transfection of the tandem plasmids into DF-1 cells significantly decreased the expression of the ALV-J envelope glycoprotein, indicating that ALV-J could inhibit plasmid replication.

### Quantitative PCR of ALV-J mRNA

Expression of the *gag *gene in cells transfected with plasmids mi-*gag*1318 and mi-*gag*1365 differed significantly from the negative control group and the null vector control group (*P *< 0.05), while expression in cells transfected with plasmids mi-*gag*1623 and mi-*gag*1971 did not differ significantly from that in the negative control and null vector control groups (*P *> 0.05) (Table 3). The use of tandem plasmids indicated significant inhibition of the expression of the ALV-J envelope glycoprotein in DF-1 cells (85-91.2%), and the rate of inhibition was highest with plasmid mi-g1318-p2516-e1384 (91.2%) (Table 4).

**Table 3:** Quantitative PCR of the miRNA-targeted gag gene inhibiting ALV-J

Group	mRNA levels of *gag*	mRNA levels of *β-actin*	mRNA levels of target gene by *β-actin *normalization	Rates of viral suppression (%)	p-values
mi-*gag*1318	1.05 ± 0.11	3.21 ± 0.12	0.327	77.3*	0.01

mi-*gag *1365	1.10 ± 0.12	3.14 ± 0.15	0.3350	65.0*	0.02

mi-*gag *1623	2.81 ± 0.11	3.49 ± 0.16	0.805	19.5	0.06

mi-*gag *1971	3.12 ± 0.17	3.85 ± 0.16	0.810	19.0	0.07

Negative control	3.15 ± 0.12	3.14 ± 0.12	1. 00	0	0

Vector control	3.62 ± 0.14	3.70 ± 0.12	0.978	0	0

**Table 4:** Quantitative PCR of the target miRNA inhibiting ALV-J replication

Group	mRNA levels of target gene	mRNA levels of *β-actin*	mRNA levels of target gene by *β-actin *normalization	Rates of viral suppression (%)	*p*-values
mi-g1318-p2516	0.62 ± 0.17	4.59 ± 0.16	0.135	86.5*	0.01

mi-g1318-e1384	0.58 ± 0.11	3.87 ± 0.13	0.150	85.0*	0.01

mi-p2516-e1384	0.48 ± 0.10	4.02 ± 0.15	0.120	88.0*	0.01

mi-g1318-p2516-e1384	0.35 ± 0.09	3.98 ± 0.12	0.088	91.2*	0.01

Negative control	3.75 ± 0.18	3.68 ± 0.15	1.030	0	0

Vector control	3.18 ± 0.12	3.25 ± 0.10	0.987	0	0

Statistically significant differences from the Negative control are indicated by*(*P *< 0.05)

## Discussion

Accumulating evidence suggests that RNAi can inhibit viral replication *in vivo *and *in vitro*. RNAi technology have been applied in numerous studies including those for HBV [11], HCV [12], HIV-1 [13], influenza virus A [14], aphthovirus of cattle [15,16], and severe acute respiratory syndrome (SARS) virus [17] infections. RNAi has been used to suppress the replication of herpesviruses, including Murine herpesvirus 68 [18], Herpes simplex virus-1 [19], Human cytomegalovirus [20], and Duck herpesvirus [21].

The ALV-J genome contains a gene arrangement of LTR-leader-*gag*-*pol*-*env*-LTR. The *gag *and *pol *genes are highly conserved, sharing 96-97% homology in ALV-J, in contrast to subgroups A, C, and D. The *pol *gene mainly encodes the reverse transcriptase (RT; P68) and viral integrase (IN; P32). RT is responsible for the production of proviral DNA using viral RNA as a template, while IN is involved in the integration of proviral DNA into the host genome. The *pol *gene is necessary for reverse transcription of the RNA genome and the generation of viral DNA, and it is a key to the insertion of the viral genome into the host genome. RNAi has been applied to the inhibition of ALV replication by a number of research groups. Chen *et al. *[8] successfully inhibited ALV-B replication induced by a retroviral vector by targeting the miRNA of the ALV-B *env *gene and its receptor encoded by the *tvb *gene.

By constructed an miRNA expression vector targeting the ALV-J *gag *gene, we demonstrated that mi-*gag*1318 and mi-*gag*1365 could significantly reduce the expression of target gene mRNA and envelope glycoprotein at a cellular level, with the highest inhibition rate of 77.3% being observed with mi-gag1318. These results showed that miRNA expression could inhibit the duplication of the target *gag *gene, with mi-*gag*1318 having the highest inhibitory effect. Accordingly, the miRNA of the *gag *target genes could successfully inhibit ALV-J replication. Thus, the successful construction of a eukaryotic expression vector would contribute to the selection and propagation of an anti-ALV related gene.

In previous studies, RNAi strategies have been successfully employed experimentally to inhibit virus replication. Hu *et al. *[7] showed that by electroporation into chicken embryos, siRNA containing ALV *gag *sequences effectively slowed down virus propagation. In the current study, the miRNA target *gag*1318 was selected that resulted in significant inhibitory effects on ALV-J replication. These miRNAs were grouped in pairs or together, and the tandem multi-target miRNAs target mi-g1318-p2516, mi-g1318-e1384, mi-p2516-e1384, and mi-g1318-p2516-e1384 were co-expressed, reducing the probability of evasion from the silencing effect of the miRNA due to virus mutation. When multiple miRNAs were expressed simultaneously, gene silencing was shown to be more effective. Tandem plasmids were transfected into DF-1 cells and then infected with ALV-J. IFA, western blotting, and quantitative PCR were used to evaluate the inhibiting effects on ALV-J replication at the cellular level. The results verified that all of the tandem plasmids could effectively inhibit the replication of ALV in DF-1 cells, with an inhibition efficiency of 85-91.2%. The enhanced inhibitory effects conferred by the multi-target miRNA expression plasmids demonstrated that the multi-target miRNAs could inhibit ALV synergistically.

These studies successfully identified targets capable of inhibiting the replication of ALV-J and are likely to be good candidates for the development of miRNA-based vaccines. The current study showed that the strategy of using multi-target miRNAs might be an effective method for inhibiting viral replication and for the acquisition of resistant mutations.

## Competing interests

The authors declare that they have no competing interests.

## Authors' contributions

QWM conceived the study, participated in its design and coordination, and finalized the manuscript in its final form. ZPZ performed the experiments. WW carried out the statistical analyses and participated in drafting of the manuscript. JT carried out the replication studies. ZGX carried out the quantitative PCR. All authors read and approved the final manuscript.
